# Genome-wide analysis of *JAZ* family genes expression patterns during fig (*Ficus carica* L.) fruit development and in response to hormone treatment

**DOI:** 10.1186/s12864-022-08420-z

**Published:** 2022-03-02

**Authors:** Miaoyu Song, Haomiao Wang, Huiqin Ma, Chuanlin Zheng

**Affiliations:** 1 College of Horticulture, China Agricultural University, Beijing, 100193 China; 2 State Key Laboratory of Agrobiotechnology, China Agricultural University, Beijing, 100193 China

**Keywords:** Fig (*Ficus carica* L.), Jasmonate, JAZ transcription factor, Hormone treatment, Expression profiles analysis

## Abstract

**Background:**

Jasmonate-ZIM domain (JAZ) repressors negatively regulate signal transduction of jasmonates, which regulate plant development and immunity. However, no comprehensive analysis of the *JAZ* gene family members has been done in the common fig (*Ficus carica* L.) during fruit development and hormonal treatment.

**Results:**

In this study, 10 non-redundant fig *JAZ* family genes *(FcJAZs*) distributed on 7 chromosomes were identified in the fig genome. Phylogenetic and structural analysis showed that *FcJAZ* genes can be grouped into 5 classes. All the classes contained relatively complete TIFY and Jas domains. Yeast two hybrid (Y2H) results showed that all FcJAZs proteins may interact with the identified transcription factor, FcMYC2. Tissue-specific expression analysis showed that *FcJAZs* were highly expressed in the female flowers and roots. Expression patterns of *FcJAZs* during the fruit development were analyzed by RNA-Seq and qRT-PCR. The findings showed that, most *FcJAZs* were significantly downregulated from stage 3 to 5 in the female flower, whereas downregulation of these genes was observed in the fruit peel from stage 4 to 5. Weighted-gene co-expression network analysis (WGCNA) showed the expression pattern of *FcJAZs* was correlated with hormone signal transduction and plant-pathogen interaction. Putative *cis*-elements analysis of *FcJAZs* and expression patterns of *FcJAZs* which respond to hormone treatments revealed that *FcJAZs* may regulate fig fruit development by modulating the effect of ethylene or gibberellin.

**Conclusions:**

This study provides a comprehensive analysis of the *FcJAZ* family members and provides information on *FcJAZs* contributions and their role in regulating the common fig fruit development.

**Supplementary Information:**

The online version contains supplementary material available at 10.1186/s12864-022-08420-z.

## Introduction

Jasmonic acid and its oxylipin derivatives (Jasmonates, JAs) are a class of lipid-phytohormones. These components participate in plant growth, development, abiotic and biotic stress responses [1, 2]. A previous study reported that JA and methyl jasmonate (MeJA) induce accumulation of protease inhibitors and adaptation mechanisms to withstand insect attack [3–5]. MeJA play a role in the initiation and modulation of fruit developmental processes by regulating JA levels [6].

Jasmonate-ZIM domain (JAZ) proteins are key repressors that negatively regulate the plant response to Jas in the signal transduction pathway. Studies report that when JAs are absent, JAZs bind to the adaptor protein novel interactor of jaz (NINJA), use the ERF-associated amphiphilic repression (EAR) domain to recruit TOPLESS (TPL) to form a co-repressor complex. They further bind to downstream transcription factors thus interfering with transcription coactivator MED25 binding [7]. On the contrary, the biologically active JA-Ile conjugate mediate JAZ protein binding to corona insensitive 1 (COI1), the F-box component of the E3 ubiquitin ligase complexes (Skip/Cullin/F-box-type, SCFCOI1), which is subsequently ubiquitinated and degraded by the 26S proteasome, thus releasing the inhibitory effect of JAZ protein on transcription factors or signal transduction components [7]. Expression patterns of *JAZs* are spatiotemporal-specific. The proteins can interact with different transcription factors, implying the functional specificity of different JAZs. In addition, they can interact with other hormone signaling pathway elements to modulate biological processes [8, 9].

JAZ family proteins are plant-specific TIFY family members. There are 13 members have been reported in Arabidopsis (*Arabidopsis thaliana*), 15 in rice (*Oryza sativa*), 23 in corn (*Zea mays*), 12 and 16 in two Petunia progenitors (*Petunia axillaris* and *Petunia inflata*) and 13 in tea plant (*Camellia sinensis*) [10–14]. Recently, Heidari done a genome-wide analysis of TIFY family genes in tomato and maize [15]. JAZ family members share a conserved TIFY sequence (TI[F/Y]XG) within the ZIM motif, which mediates interaction of JAZ and NINJA or is implicated in dimerization of JAZ-JAZ proteins. MYC2 is a basic helix-loop-helix-like transcription factor that binds the G-box-containing promoters of the downstream genes, and interacts with MED25 subunit [16, 17]. The C-terminal Jas domain is implicated in interaction of JAZs and MYC2, thus suppressing JA responses and distinguishes JAZs members from the other members of the TIFY family (such as PEAPOD subfamily) [16, 18–20]. In the ubiquitin JAZ-mediated degradation pathway, the Jas domain facilitates interaction between JAZ and COI1, with the conserved motif of SLX2FX2KRX2RX5PY [21]. ZIM and Jas domains are highly conserved among the JAZ protein members, indicating there are functional importance and synergy among the JAZ family members [8]. Single gene loss-of-function mutant plants of JAZ5 or JAZ13 do not show significant difference compared with the wild type plant. On the contrary, JAZ5/10 and JAZ10/13 double mutants are significantly sensitive to JA application. Furthermore, the ten JAZ mutant plant jazU (JAZ1/2/3/4/5/6/7/9/10/13) showed more significant phenotype compared with the five mutant jazQ (JAZ1/3/4/9/10), including severe growth inhibition and high sensitivity to JA application [22].

Members of JAZ family interact with different transcription factors (MYC/JAM/ICE/MYB/TOE) to regulate specific downstream genes expression, involved in regulation of root elongation and growth [23], leaf senescence [24], insect resistance [25], freezing stress [26, 27], flowering time of plants [28]. Moreover, JAZs participate in regulation of various biological processes, by combining with the components of the other hormone signal transduction pathways [8]. For example, Arabidopsis JAZ protein can interact with ethylene insensitive3 (EIN3) and EIN3-LIKE1 (EIL1), the core transcription factors in the ethylene signaling pathway, and interfere with their activity in root growth regulation [29]. DELLA protein, the inhibitor of gibberellin (GA) signal transduction, can also interact with JAZ protein thus regulating the balance between plant growth and development, and defense responses [30].

Common fig (*Ficus carica* L.) is one of the oldest horticultural crops found in many parts of the world. Fig fruit has antioxidant properties with pleasant taste and high nutritional value [31]. Fig fruit is an aggregate fruit consisting small single drupes. The edible part develops from a closed ovary, called syconium, which contains many unisexual female flowers [32]. During the process of fig fruit development, a typical double sigmoid growth curve is observed, known as fast-slow-fast growth period [33]. After long-term evolution, common fig has established a complex resistance mechanism to resist stress by abiotic factors, pathogens and insects. JAs are reported to play an important role in this regulation [34]. However, genome-wide characterization of the JA signaling-related components and their expression patterns in fig fruit has not been performed. In the current study, whole-genome data of “Horaishi” and “Dottato” [35, 36] and transcriptome data of “zibao” fig from different developmental stages (NCBI accession No. PRJNA723733, data uploaded by our team) were used for prediction of the *JAZ* family members. Characteristics of *FcJAZs*, genome-wide gene co-expression network and expression patterns were analyzed. The findings provide understanding of the roles of *FcJAZs* in regulating fig fruit development.

## Results

### JAZ family members were identified in fig

HMM profile and protein blast analyses were performed to identify JAZ members in fig. There are 10 JAZ proteins were identified from the fig genome and were named FcJAZ1 ~ FcJAZ10 (Table 1). Analysis using InterProScan and SMART webservers indicated that all of the 10 putative JAZ proteins had highly conserved TIFY and Jas domains. The CDS lengths of the 10 *FcJAZ* genes ranged from 381 bp (*FcJAZ7*) to 1155 bp (*FcJAZ3*). *FcJAZs* genes were distributed in 7 chromosomes. The theoretical molecular weights ranged from 14,163.97 Da (FcJAZ8) to 42,241.05 Da (FcJAZ7) and isoelectric points (pI) of these proteins ranged from 4.92 (FcJAZ5) to 9.81 (FcJAZ7). All predicted proteins showed basic pH (pI > 7) apart from FcJAZ5. Prediction of protein subcellular localization indicated that all FcJAZs proteins were localized in the nucleus.

**Table 1:** The properties of the 10 identified JAZ family members in fig

Gene Name	ID	CDS (bp)	Chromosome	Chr start	Chr end	Strand	Molecularweight (Da)	Theoretical pI	Identity	Location
FcJAZ2	c66235_g1	1,047	Chrom 6	12,122,436	12,124,768	-	39,949.91	9.52	0.739	nuclear
FcJAZ3	c78226_g2	1,155	Chrom 11	10,036,175	10,038,886	+	42,241.05	8.97	0.739	nuclear
FcJAZ4	c39574_g2	612	Chrom 11	3,746,873	3,748,764	+	22,505.50	9.35	0.739	nuclear
FcJAZ5	c1342_g1	612	Chrom 9	11,307,798	11,309,907	+	22,520.05	4.92	0.478	nuclear
FcJAZ6	c43020_g4	882	Chrom 1	16,323,905	16,325,349	+	31,758.05	9.55	0.609	nuclear
FcJAZ7	c45700_g1	381	Chrom 5	18,376,930	18,378,581	-	14,423.58	9.81	0.522	nuclear
FcJAZ8	c32102_g1	390	Chrom 6	10,695,805	10,696,529	-	14,163.97	9.38	0.391	nuclear
FcJAZ9	c22895_g1	1,050	Chrom 8	10,091,529	10,094,861	-	38,047.37	8.43	0.522	nuclear
FcJAZ10	c45035_g1	960	Chrom 10	3,940,043	3,942,696	+	36,516.28	9.56	0.217	nuclear

We identified 21 duplicate gene pairs in the JAZ family of figs (Supplementary Table 4). The Ka/Ks ratios of FcJAZ1/FcJAZ10, FcJAZ1/FcJAZ7, FcJAZ1/FcJAZ3, FcJAZ6/FcJAZ2, FcJAZ9/FcJAZ10, FcJAZ10/FcJAZ8, FcJAZ10/FcJAZ2, FcJAZ4/FcJAZ2 and FcJAZ5/FcJAZ2 were > 1, suggesting positive selection. The Ka/Ks ratio of the remaining 12 pairs were < 1, indicating that they are negatively selected. Negative selection is redundant with positive selection and (Ka/Ks < 1) indicates that harmful mutations are eliminated in the gene family and the protein remains unchanged, which is a purification selection.

### Phylogenetic analysis and sequence alignment of FcJAZs showed high conservation of JAZs proteins

**Fig. 1:**
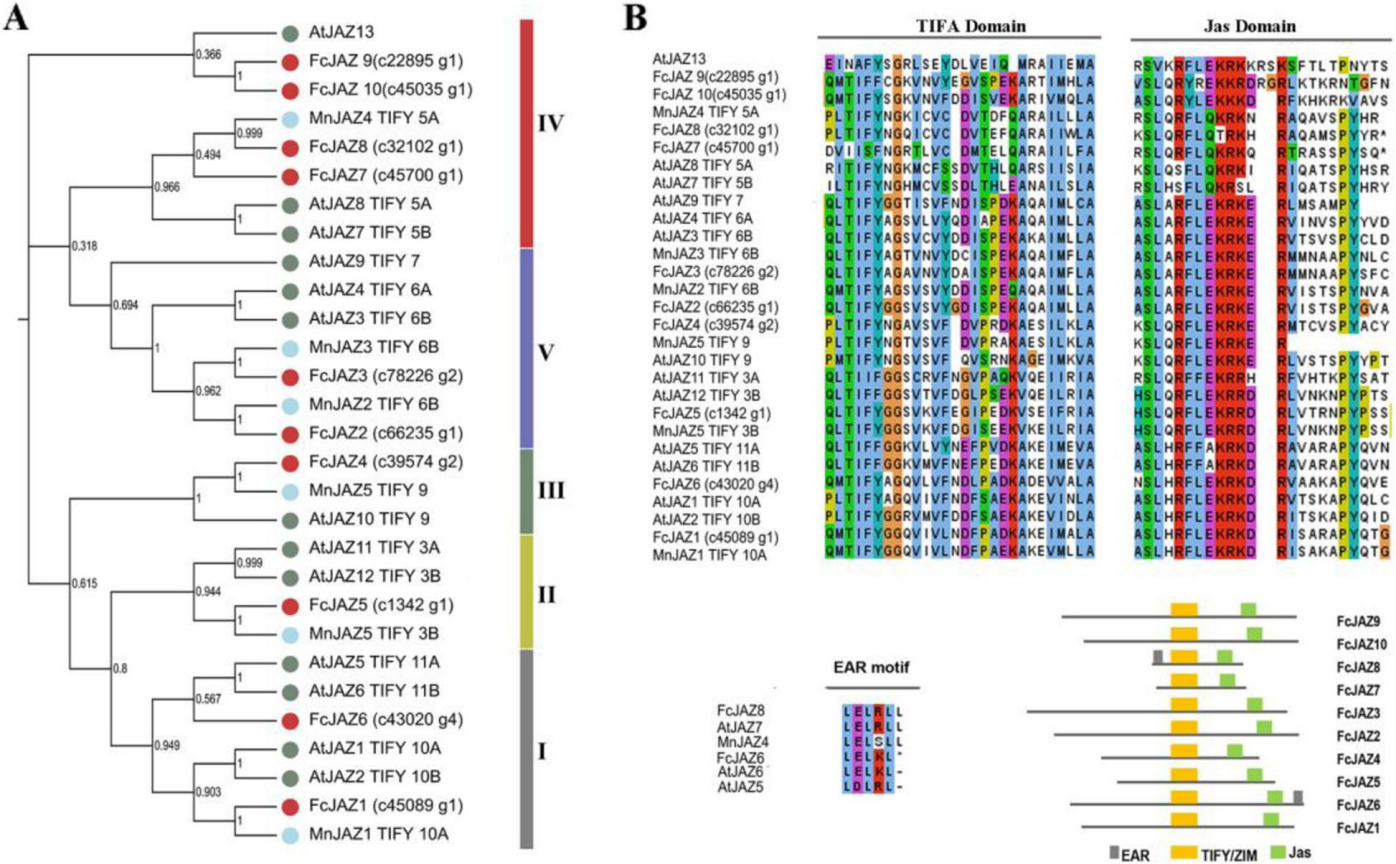
Phylogenetic analysis and conserved motif of FcJAZs in fig.** a** The phylogenetic relations of fig (10), mulberry (6) and Arabidopsis (13) JAZ family members were performed in the software of MEGA 6.0, using the neighbor-joining method with 1000 bootstrap replicates. Bootstrap scores were labelled on the nodes and the 10 members of FcJAZs were marked with red color. See the accession number of these proteins in the supplementary Fig. 1. **b** Distribution of the conserved motifs of FcJAZs. Sequence alignment of the conserved TIFY domain, Jas motif and EAR motif were performed by CLUSTALW among fig mulberry and Arabidopsis.

To explore the evolutionary relationships among the FcJAZ proteins, ML tree of mulberry, Arabidopsis and fig JAZs proteins were built using MEGA 6.0. Accuracy of tree branching was determined through bootstrap analysis with 1000 replicates. The findings showed that mulberry, Arabidopsis and fig JAZs grouped in five well-defined subfamilies (Fig. 1A). Analysis of JAZ proteins relationship showed a closer relationship between fig and mulberry proteins compared with fig and Arabidopsis proteins.

TIFY (acc. PF06200) and jas domain (acc. PF09425) conserved motifs were explored in all FcJAZ proteins (Fig. 1 B). Motif analysis was performed using MEME webserver to identify the conserved domain in these proteins. The findings showed that ZIM domain of TIFY contained a conserved TIFYXG motif, and the core sequence of the jas domain was SLXRFLEKRX(2)R (Fig. 1 B; Fig S1). Notably, the N-terminus of FcJAZ8 and the C-terminus of FcJAZ6 contained an EAR domain (LxLxLx type), which is implicated in repressing transcription through interaction with the TOPLESS (TPL) co-repressor (Fig. 1 B; Fig S1) [21]. Multiple sequence alignment of the protein sequences indicated that the Jas domain contains the degron sequence (LPIARR) which plays an important role in COI1–JA-Ile–JAZ complex formation and is physically associated with JA-Ile (Fig S1).

### *cis*-elements and gene structure analysis of *FcJAZs*

**Fig. 2:**
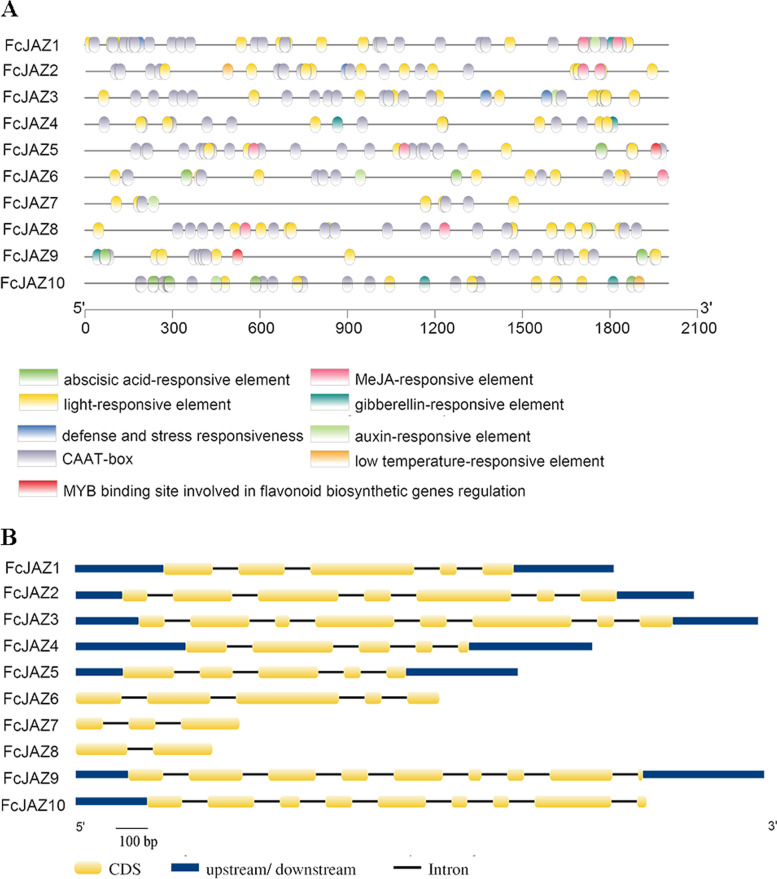
Analysis of *cis*-elements and gene structures for *FcJAZ* genes. **a** The distribution of *cis*-elements in the 2000 bp upstream promoter regions of *FcJAZ* genes were analyzed. The *cis*-elements were predicted at PlantCARE (https://bioinformatics.psb.ugent.be/webtools/plantcare/html/) and PlantPAN (https://plantpan.itps.ncku.edu.tw/). Different *cis*-elements were presented with different shapes and colors. **b** Analysis of exon–intron structures for *FcJAZ* genes. Blue rectangles, yellow rectangles and blank lines were indicated upstream/downstream, exons and introns, respectively. The sizes of exons and introns could be estimated by the scale below

To explore the gene structure and evolutionary trajectory of *FcJAZs*, *cis*-elements located in the promotor region and organizations of exon–intron were analyzed. *Cis*-elements analysis was performed using the 2000 bp region upstream of the *FcJAZ* genes using PlantCARE and PlantPan tools. The findings showed that most of the promoters in *FcJAZs* contained various plant hormone response-related elements (Fig. 2 A). In addition to MeJA, abscisic acid (ABRE), Auxin (TGA and HD-Zip 3), gibberellic acid (P-box, TATA box and CARE), ethylene (W-box) and salicylic acid (TCA) related elements were identified in the promotor regions. Notably, light-responsive elements were the most abundant *cis*-elements. *FcJAZ5* and *FcJAZ9* contained a MYB binding site [aC(G/C)GTTA] which is implicated in flavonoid biosynthesis (Table S2) [37]. High intron number correlates with complex regulation, implying a more recent origin of the gene [38]. Analysis showed presence of 1–8 introns in *FcJAZ* genes (Fig. 2B). *FcJAZ8* had lowest number of introns, whereas *FcJAZ9* and *FcJAZ10* had the highest number of introns. Notably, a high sequence similarity observed in the exon regions implies a complex regulatory during evolutionary process.

For the fig fruit development, the RNA-seq data for *FcJAZ* genes expression patterns were obtained from the NCBI database (Accession No. PRJNA723733), and further verification of the RNA-seq results were performed by the qRT-PCR. The red line indicated the qRT-PCR results, and the blue line indicated the RNA-seq results. For the different tissues, the expression levels *FcJAZ* genes were determined by qRT-PCR, different shapes and colors represent different tissues as indicated in the figure. **a** FcJAZ family members with FPKM value greater than 100 in RNA-seq results. **b** Members of the FcJAZ family with FPKM between 0–100 in the RNA-seq results. Different letters indicated significant differences according to Duncan’s new multiple range test for the qRT-PCR (red) and RNA-seq (blue) data respectively, *P* < 0.05.

### Expression analysis of *FcJAZs* in development stage and different tissues

**Fig. 3:**
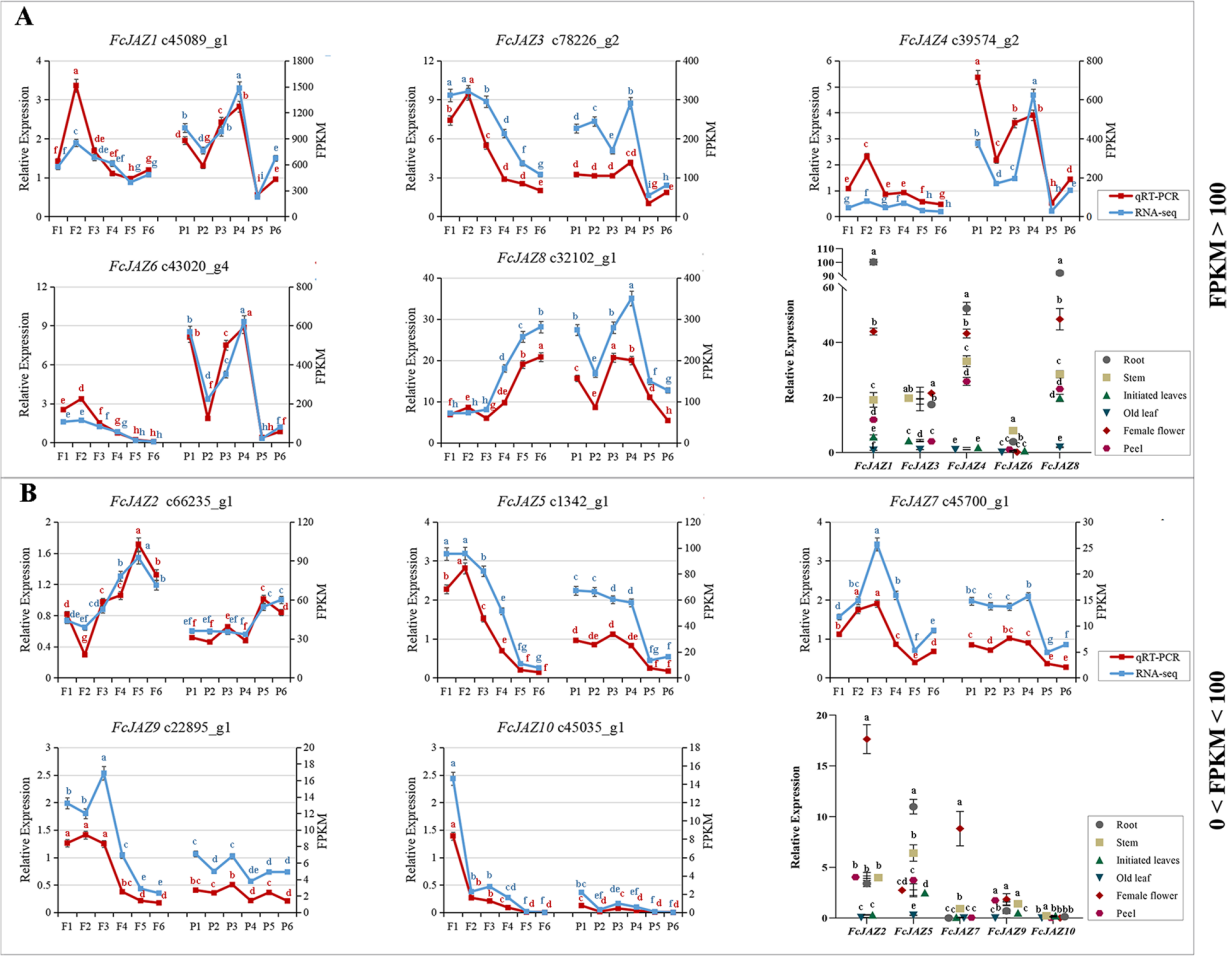
*FcJAZ* genes expression pattern during the fig fruit development and different tissues

To explore the expression pattern of *FcJAZ* genes during the developmental process of fig fruits, female flower and fruit peel were collected at different stages of growth. RNA isolation and gene expression analysis was then performed using the female flower and fruit peel samples. Analysis of female flower development showed that most *FcJAZ* genes were expressed at a high level in the early stages then expression levels decreased in the later stages. Analysis of expression profiles of *FcJAZ2* and *FcJAZ8* revealed lower expression level in the early stages than in later stages (Fig. 3). Analysis of fruit peel development showed that most of *FcJAZs* had low expression levels in the later stages whereas *FcJAZ2* was slightly upregulated in the later stages. Most *FcJAZs* were significantly downregulated from stage 3 to 5 in the female flower whereas most genes were upregulated from stage 4 to 5 in the fruit peel. These findings indicate that these regulated *FcJAZ* genes may play significant roles at special windows during the process of fig fruit development.

To examine the expression level of *FcJAZ* genes in specific tissues, root, stem, young leaf, old leaf, female flower and fruit peel of fig tree was sampled and used for RNA analysis and gene expression analysis. The findings showed that the expression patterns of *FcJAZ1*, *FcJAZ4* and *FcJAZ8* were similar in the group of FPKM > 100. *FcJAZ1*, *FcJAZ4* and *FcJAZ8* were highly expressed in the root, followed by female flower and stem. *FcJAZ3* was highly expressed in the female flower, stem and root in that order whereas *FcJAZ6* was highly expressed in stem and root (Fig. 3A). In the group of 0 < FPKM < 100, *FcJAZ2* and *FcJAZ7* genes were highly expressed in the female flower, *FcJAZ5* was highly expressed in the root, and the other *FcJAZ*s showed low expression levels in these six tissues (Fig. 3B). These findings indicate that *FcJAZ* genes have different expression patterns during the process of fig fruit development and show tissue specific expression.

The RNA-seq data for the fig fruit development were used for the WGCNA analysis, which performed by the WCGNA package (version 1.63) of the R software and the analysis conditions as indicated in the Materials and Methods. Genes within different modules were labeled with different colors according to the WGCNA's conventions. The correlation results in female flower were represented in (**a)** and in the fruit peel were represented in **(b)**. The number on the up of each cell illustrated the corresponding to the correlation coefficient and the number in bracket were the *P*-value. High correlation coefficients are presented in red color whereas the low correlation coefficients are presented in blue color.

### Weighted gene co-expression network analysis (WGCNA) of *FcJAZs* during the fig fruit development

**Fig. 4:**
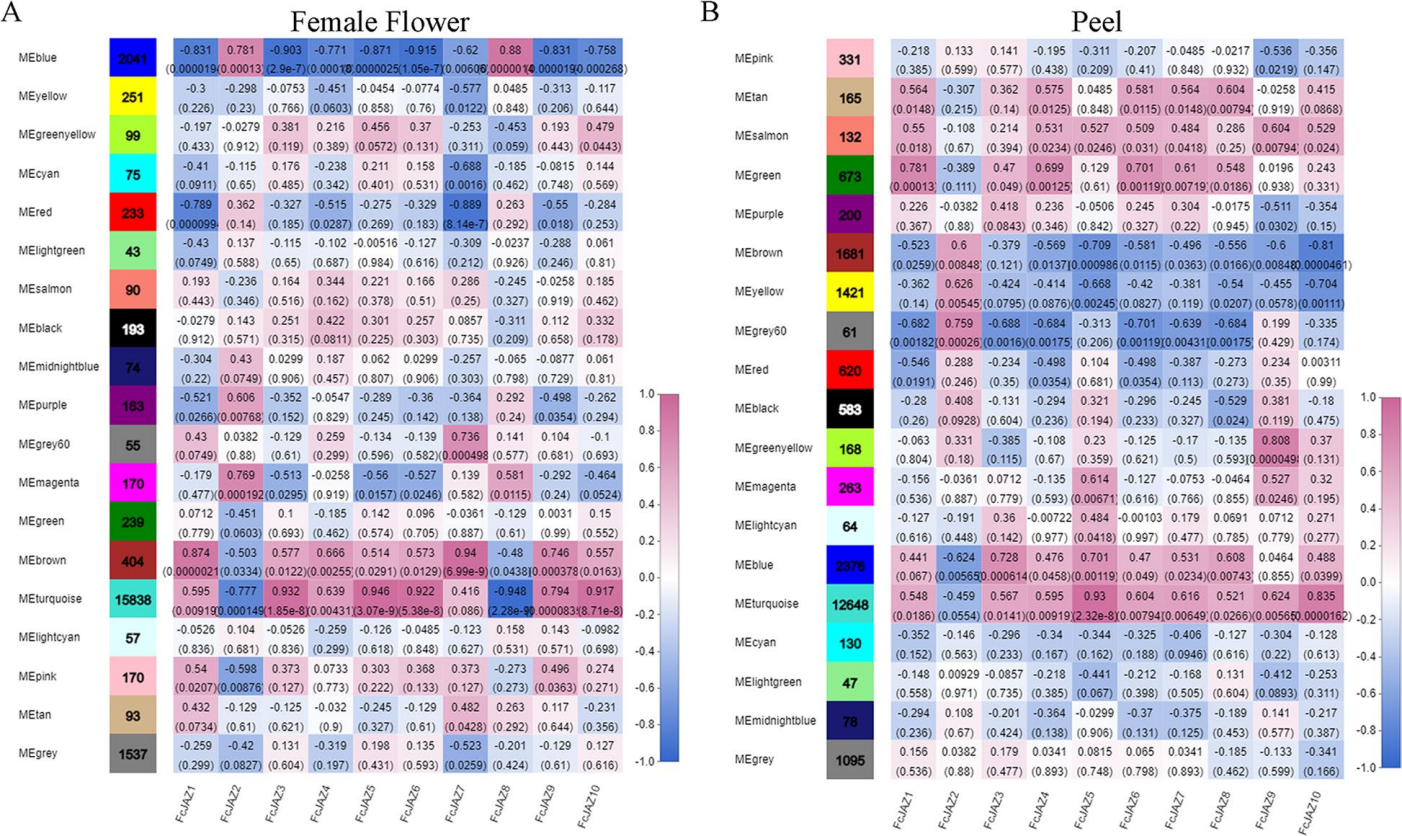
Co-expression network analysis (WGCNA) of *FcJAZ* genes during the fig fruit development

The results of WGCNA above and previous RNA-seq data (NCBI number PRJNA 723,733) were used to interpret the correlation pattern between FcJAZs and the development of fig tissue. Genes with similar expression patterns were clustered in an unbiased manner. After calculating and matching patterns related to the *FcJAZs* expression patterns in fig female flower and peel, highly related genes were grouped into clusters (modules). WGCNA showed that 21,825 fig genes (FPKM ≥ 10) were grouped into 19 modules and different colors were assigned to name each module (Fig. 4). Three modules including Brown, turquoise and pink were positively correlated with most of *FcJAZs* expression patterns (correlation coefficient > 0.5, P value < 0.05) the female flower, and 16,412 genes (Fig S2 A). Four modules, blue, yellow, red and purple were negatively correlated with *FcJAZs* expression patterns (correlation coefficient < -0.5, P value < 0.05) in the female flower and 2,688 genes were obtained (Fig S2 A). On the contrary, *FcJAZ2* and *FcJAZ8* showed opposite regulatory correlations with the other 8 FcJAZs. These findings imply that these two genes may play a role as inhibitors during female flower development. Analysis of the peel showed that six modules including tan, salmon, green, purple, blue and turquoise were positively correlated with *FcJAZs* expression patterns, and 16,194 genes were screened out. Four modules including brown, yellow, grey60 and red were negatively correlated with *FcJAZs* expression patterns, and 3,783 were screened out. Notably, *FcJAZ2* showed significant differential regulation in the peel compared with the other genes (Fig S2 A). There are 12,158 genes showed positive or negative correlations with *FcJAZs* in the female flower and peel. KEGG analysis was performed for these genes. During fruit development, the expression trend of JAZs in female flower and peel was mainly clustered into plant hormone signal transduction (87 genes) and plant-pathogen interaction (98 genes). (Fig S2 B). There are 1,547 genes showed diverse trends, and were mainly enriched in flavonoid biosynthesis, phenylpropanoid biosynthesis, phenylalanine metabolism, and flavone and flavonol biosynthesis pathway (Fig S2 B, C).

### Interactions among FcJAZs and between FcJAZs with FcMYC2

**Fig. 5:**
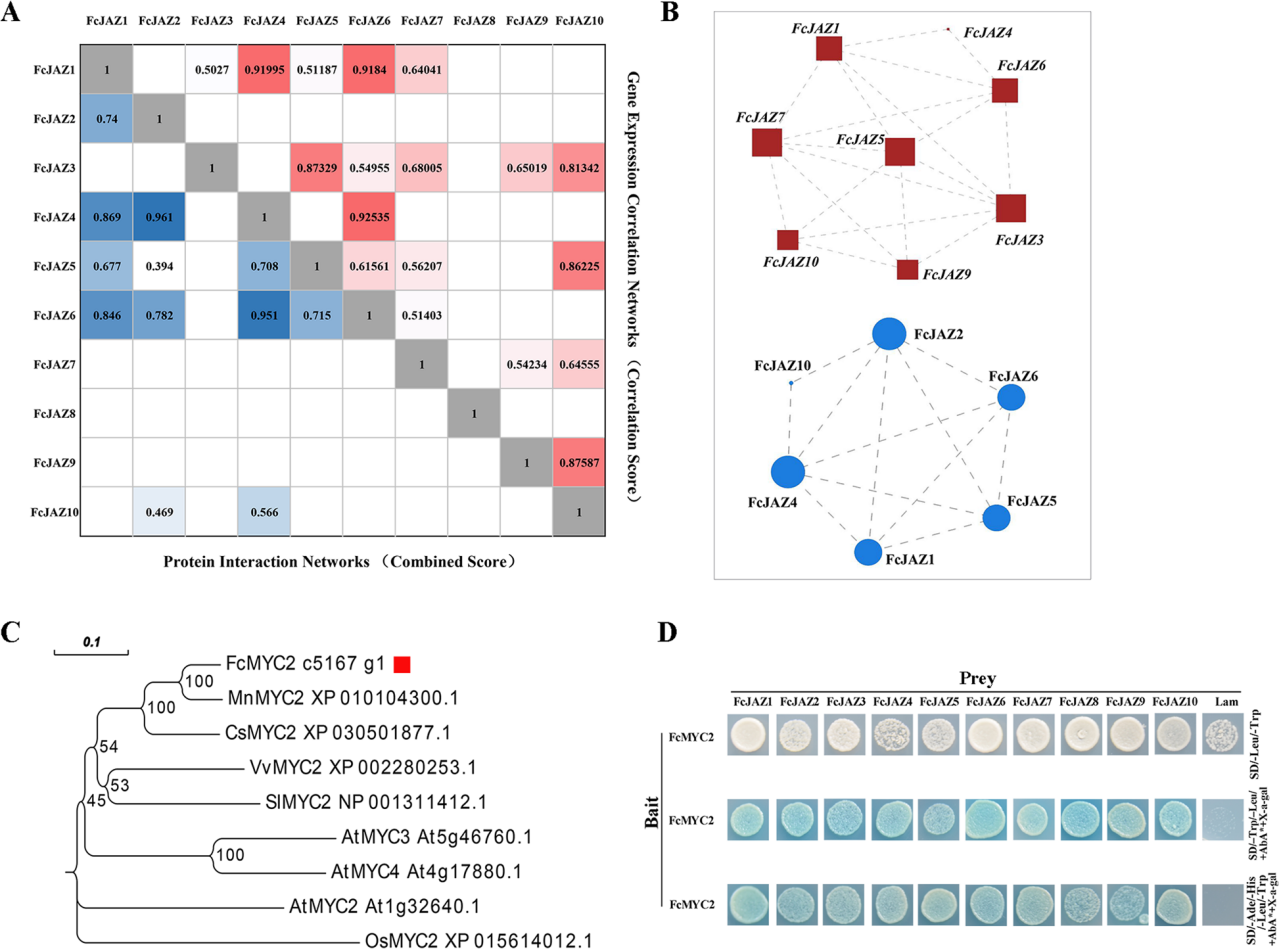
**C**orrelation analysis among FcJAZs-FcJAZs and FcJAZs-FcMYC2.** a** The *FcJAZs*-*FcJAZs* gene expression correlation analysis were performed by pearson algorithm, based on these gene’s expression of female flower and peel during the fig fruit development, the data in the cells with red color were indicated the correlations scores. And the FcJAZs-FcJAZs proteins interaction network were analyzed in the STRING database, the data in the cells with blue color were indicated the required confidence (combined score). **b** The co-regulatory network of FcJAZ was illustrated by Cytoscape. The augmented icon displayed higher reliability. **c** Phylogenetic analysis was performed tree that shows the homology of each MYC2 sequences by MEGA 6.0 using the neighbor-joining method with 1000 bootstrap replicates. **d** Y2H assay were used to analyze the interaction between FcJAZ proteins and transcription factor FcMYC2. A combination of recombinant pGBKT7-MYC2 (bait) and corresponding to pGADT7-FcJAZ constructs (prey) were co-transformed into yeast strain Y2H, and further plated on DDO (SD/-Leu/-Trp) medium as control, or on SD/-Ade/-His/-Leu/-Trp/ + X-a-Gal/ + AbA* medium to test the protein interactions

JAZ family members can form homodimers or heterodimers through interaction at the ZIM domain to regulate downstream gene expression and exhibit their biological functions [39]. *JAZs*-*JAZs* gene expression correlation networks were analyzed based on expression of the genes during the development stage of fig fruit (RNA-seq data was used in this analysis as described in Materials and methods section). The correlation was presented in heat maps. Protein–protein interaction networks were constructed using STRING tool. The top three co-expression pairs were *FcJAZ4*-*FcJAZ6*, *FcJAZ4*-*FcJAZ1* and *FcJAZ6*-*FcJAZ1*; whereas the top four pairs with high values in the protein–protein interaction network were FcJAZ2-FcJAZ4, FcJAZ4-FcJAZ6, FcJAZ1-FcJAZ4 and FcJAZ1-FcJAZ6. The top three co-expression genes were part of the top four protein–protein interaction network (Fig. 5A). Further analysis showed that, besides *FcJAZ2* and *FcJAZ8*, the other eight members showed high correlation. *FcJAZ5* showed the highest correlation with the other five *FcJAZs* with a co-expression score above 0.5. Notably, *FcJAZ5* was located at the center of the co-expression network. Analysis of the protein interaction network showed that although FcJAZ2 had no significant correlation with other members in the network, it had a higher protein interaction coefficient with four FcJAZs (FcJAZ4, FcJAZ5, FcJAZ6 and FcJAZ10). FcJAZ8 did not show interaction with the other FcJAZs, and did not form dimers with the other FcJAZs (Fig. 5B). FcJAZ6 protein contained EAR motif, thus FcJAZ1 and FcJAZ4 may perform their functions by recruiting FcJAZ6 to form heterodimers.

The C-terminal Jas domain of JAZ plays an important role in interaction of JAZ-JAZ or JAZ-MYC2 [16]. NCBI CD-search was used to predict a MYC2 like gene—FcMYC2 from the fig genome. This gene had the conserved domain of bHLH-MYC_N, which mediates interaction with the JAZ repressor, and HLH DNA-binding domain which mediates heterodimerization of JAZs (Figure S3A) [40]. Sequence alignment showed that the basic domain was highly conserved between FcMYC2 proteins and other MYC2s proteins (Figure S3B). Phylogenetic analysis showed that FcMYC2 was highly homologous to AtMYC2 (*Arabidopsis thaliana*), MnMYC2 (*Morus notabilis*), CsMYC2 (*Citrus sinensis*) and VvMYC2 (*Vitis vinifera*) (Fig. 5C). Y2H was used to perform interaction analysis of FcJAZs with FcMYC2. The finding showed that all of the ten combinations used for transformation of yeast grew normally on the plate of DDO (SD/-Leu/-Trp) + X-a-Gal/ + AbA. The clones were further incubated on SD/-Ade/-His/-Leu/-Trp/ + X-a-Gal/ + AbA medium. All FcJAZs-FcMYC2 combinations showed growth, whereas the negative control did not grow, implying that all the ten FcJAZs directly interact with FcMYC2 (Fig. 5D).

The results showed that, besides the combinations of FcJAZ7/FcMYC2 and FcJAZ8/FcMYC2, all the rest 8 combinations of FcJAZs-nYFP and cYFP-FcMYC2 could detect a strong fluorescence signal, suggesting they may have directly interaction between them. (Figure S5).

The expression pattern of *FcJAZs* and *FcMYC2* in response to 200 μM MeJA treatment were determined by qRT-PCR in fig female flower and fruit peel. The details of the treatment were described in the Materials and Methods. The bar indicated the control treatment and the line indicated MeJA treatment. Values of qRT-PCR represent the mean ± SD of three biological replicates.

* indicated significant difference from control according to Student’s *t* test, *P* < 0.05.

### Expression pattern of *FcJAZs* and *FcMYC2* in response to MeJA

**Fig. 6:**
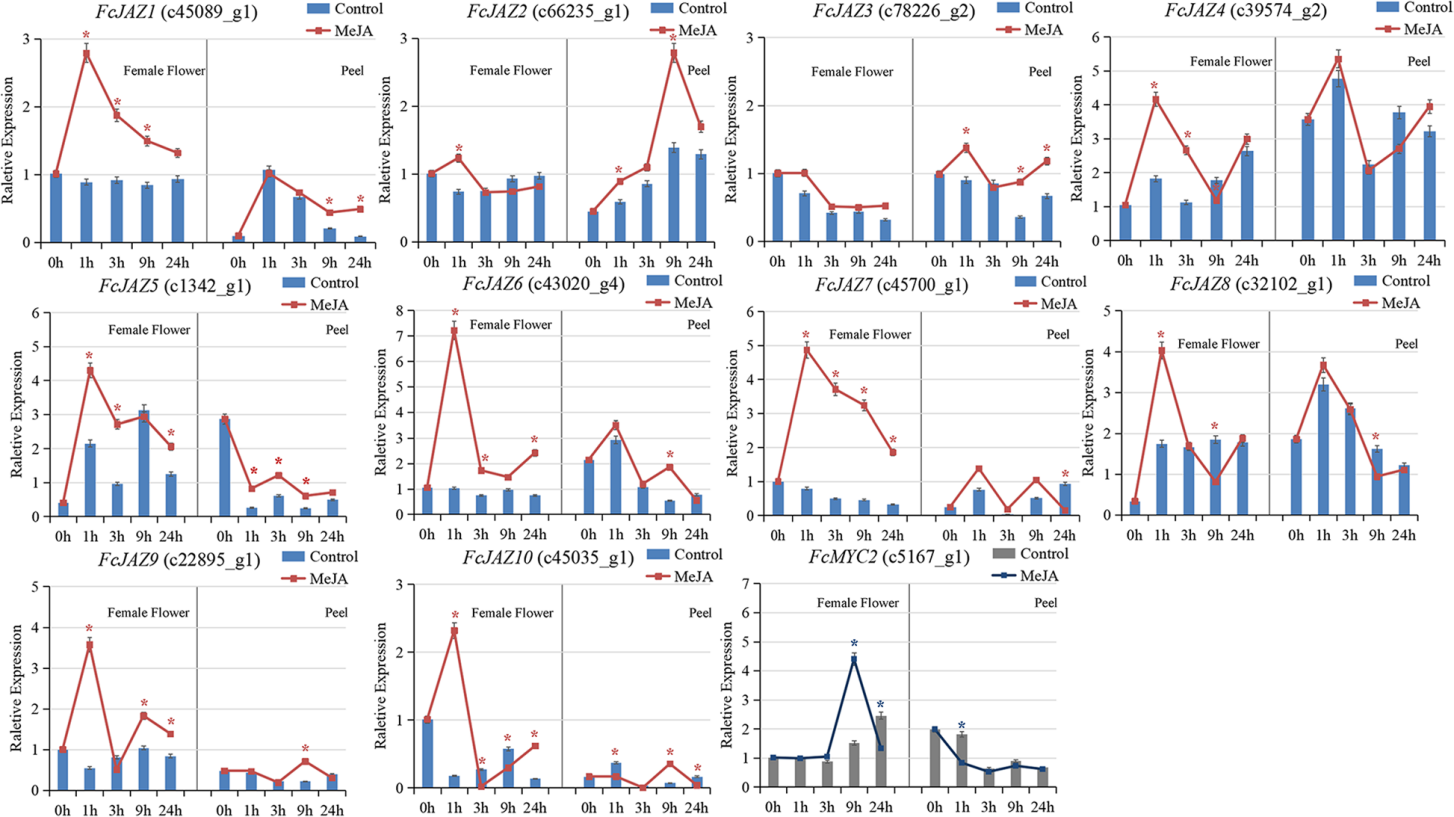
MeJA application modulated the expression pattern of *FcJAZs* and *FcMYC2* in fig fruit

To explore the response of *FcJAZs* and *FcMYC2* to JA application, the female flower and peel of fig were treated with MeJA. Gene expression analysis showed that most *FcJAZs* were upregulated in female flower tissues at 1 h after treatment compared with control treatment (Fig. 6). *FcJAZ6* and *FcJAZ7* were the most upregulated *FcJAZs* genes at 1 h after MeJA application, showing 6.96 and 6.35 fold upregulation, respectively. Induced expression of *FcJAZ2* and *FcJAZ3* were not significant in the female flower. Analysis of the peel showed that the effect of MeJA application was not significant compared with that in the female flower. *FcJAZ2, FcJAZ6* and *FcJAZ9* showed significant upregulation at 9 h after treatment, whereas the other *FcJAZs* showed insignificant upregulation. The expression pattern of *FcMYC2 was* positively correlated with MeJA application in female flowers at 9 h, whereas *FcMYC2* was downregulated in the peel at 1 h. These findings indicate that the female flower of fig was more sensitive to MeJA compared with the fig peel.

For the effect of Ethephon and GA_3_ on *FcJAZs* expression in fig fruit, the RNA-seq data were obtained from the NCBI database (Accession No. SRP115264 and No. PRJNA606407) (Chai et al., 2018; Cui et al., 2020). The expression pattern of *FcJAZs* responded to ethephonG3 application were represented in **(a)** and **(b)**, corresponding to the fig female flower and peel; and the expression pattern of *FcJAZs* responded to GA_3_ application were represented in **(c)** and **(d)**, corresponding to the fig female flower and peel. The FPKM data of *FcJAZs* were represented in the red cells. The regulation of up and down were represented as blue and red circles, respectively. F, female flower tissue; P, peel tissue.

### Expression patterns of ***FcJAZs*** in response to ethyphon and GA_3_

**Fig. 7:**
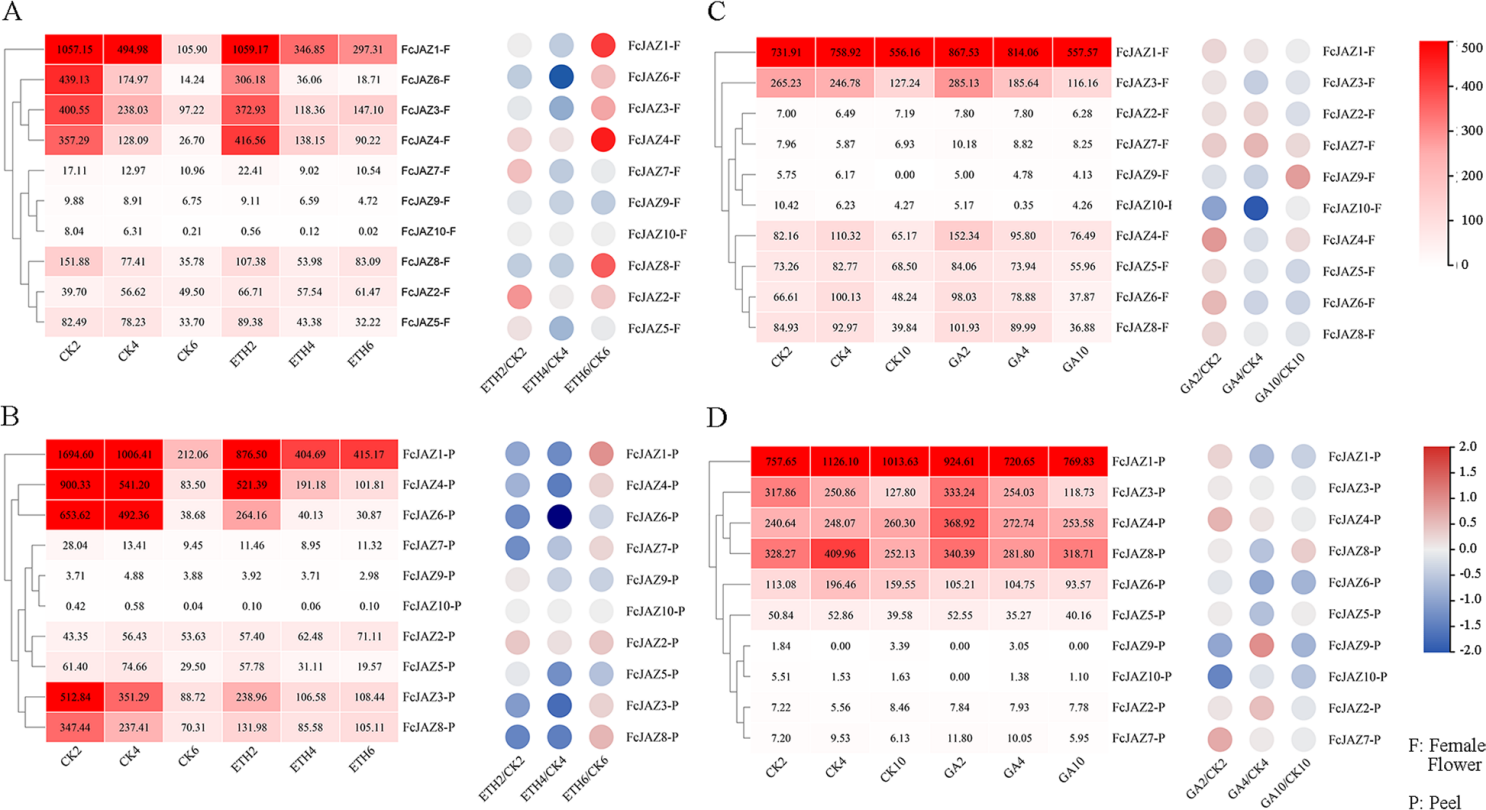
Ethephon and GA_3_ modulated the expression patter of *FcJAZs* in fig fruit

To explore *FcJAZs* responses to ethyphon and GA_3_ application in fig fruit, related expression datasets of *FcJAZs* were retrieved from NCBI GEO datasets (Accession No. SRP113799 and No. PRJNA606407) [41, 42]. Analysis showed that opposite responses of *FcJAZs* expression profiles between fig female flowers and peels (Fig. 7A, B). In the early stage at day 2 or 4 after ethyphon treatment, the findings showed that *FcJAZ 1* and *FcJAZ 3–8* were significantly downregulated (≥ twofold, q = 0.05) either at one of these two time points or at the two time points in the peel. However, analysis did not significant change for *FcJAZ2* and *FcJAZ 9–10* at these two time points. Analysis of the female follower showed that the main significant modulations were on *FcJAZ1*, *FcJAZ3*, *FcJAZ4* and *FcJAZ8*, which were significantly upregulated at day 6 after ethyphon treatment. Notably, *FcJAZ6* was the only significantly downregulated gene after ethyphon treatment, whereas other genes were not significantly modulated by ethyphon. Analysis showed less regulation of *FcJAZs* by GA_3_ treatment compared with ethyphon treatment. *FcJAZ6* and *FcJAZ10* were downregulated after GA_3_ application in peels whereas *FcJAZ4* and *FcJAZ7* were upregulated. Analysis showed that *FcJAZ9* was downregulated at day 2 and upregulated at day 4 after GA_3_ treatment of female flowers. Notably, *FcJAZ10* was downregulated at day 4 after GA_3_ application, *FcJAZ4* and *FcJAZ9* were up regulated after GA_3_ treatment, whereas GA_3_ treatment did not significantly affect expression of the other genes (Fig. 7C, D).

## Discussion

JA is an important hormone widely involved in plant growth, development, and abiotic and biotic stress [1, 2, 29]. JAZ are the key components of JA signal transduction pathway. JAZ plays a role as a repressor and negatively regulates plant responses to JA. JAZs have been reported in some important agricultural crops, such as grape [43], maize [18], bread wheat [44], tomato [45], strawberry [46] and tea [14]. However, no comprehensive analysis has been conducted on the JAZ protein members in common fig.

### Structural characteristics and evolution of the JAZ gene family in fig

There are 10 JAZ family members were isolated in figs.They were FcJAZ1 (c45089_g1), FcJAZ2 (c66235_g1), FcJAZ3 (c78226_g2), FcJAZ4 (c39574_g2), FcJAZ5 (c1342_g1), FcJAZ6 (c43020_g4), FcJAZ7 (c45700_g1), FcJAZ8 (c32102_g1), FcJAZ9 (c22895_g1) and FcJAZ10 (c45035_g1) (Table 1). All the FcJAZs had the conserved TIFY/ZIM domain and Jaz motif. The core sequence of TIFY motif has different compositions in other plants, such as VIF[F/Y]XG, TLF[F/Y]XG, TLL[F/Y]XG, TLV[F/Y]XG, TMF[F/Y]XG, TII[F/Y]XG and TIS[F/Y]XG [47]. The TIFY motif in the core sequence of most FcJAZs comprised TIFYXG, whereas in FcJAZ9 it comprised TIFFXG and IISFXG in FcJAZ7. This finding indicates diversity of the core TIFY motif structure, which may be correlated with different functions of JAZs. The sequence of the Jas domain is relatively conserved among plants, including figs. Jas domain plays an important role in regulation of JA sensitivity, perception and interaction with transcription factors [10]. In addition, FcJAZ6 and FcJAZ8 displayed EAR type motif that may be implicated in binding of TPL to repress JA responses without the presence of NINJA [19]. This indicates that JAZ proteins with EAR motif may play a role as negative feedback regulators to inhibit the jasmonic acid signaling pathway, thus modulating plant growth, development and defense response [19].

Analysis of the subfamilies based on the characteristics of the motif showed that FcJAZ2 and FcJAZ3 belonged to the V subfamily. In addition, FcJAZ2 and FcJAZ3 were mainly found in the early stage of the evolutionary process, which contained the typical ZIM and Jas conserved domains [48]. These two genes may be involved in plant growth, reproduction, senescence, hormone synthesis and defense response [49]. Although FcJAZ1 and FcJAZ6 grouped in subfamily I contained the typical ZIM and Jas conserved domains, the EAR motif was identified at the C-terminus of FcJAZ6. This finding implies that this family members may have appeared in latest stage of the evolution process, and may be involved in some plant-specific biological processes (Fig. 1).

JAZ genes structures are diverse in plants as mainly reflected in the length and number of introns [12]. Shorter introns are more often retained, whereas longer introns are mostly alternately spliced [50]. Analysis did not show significant differences in lengths of intros in the *FcJAZs* of fig, which may can be attributed to long-term selection pressure. Members of the JAZ gene family mainly contain 0–7 introns in some typical plants, such as rice, wheat and Arabidopsis. The number of introns in *FcJAZs* were 1 to 8. *FcJAZ9* and *FcJAZ10* in subfamily IV showed the highest number of introns (8), whereas *FcJAZ8* had only 1 intron and *FcJAZ7* had two introns, and all belonged to subfamily IV. Notably, *JAZ* genes with fewer introns within the same subfamily respond faster to environmental stress compared with *JAZ* genes higher number of introns [44, 51]. Therefore, *FcJAZ8* may play an important role in fast response to environmental stress.

### Expression profiles of *FcJAZs* during fig fruit development and in response to hormone treatments

JAZ negatively regulates signal transduction of JA. JAZ regulatory functions are mainly dependent on gene expression level and location in specific tissues [52]. The findings of the current study showed that, *FcJAZs* genes with either high (> 100) or low (0 < FPKM < 100) FPKM value were mainly expressed in the female flower. This implies that FcJAZs have an important role in modulating JA signal transduction during fig fruit development.

Therefore, expression profiles of *FcJAZ* genes were analyzed during the fig fruit development stage and in response to hormone treatments.During the natural process of plant development, plants exhibit immune response through the JA signaling pathway when they encounter biotic or abiotic stress. This response may occur through repression of vegetative growth and promotion of the reproductive development under these situations [28, 53]. The fig plant may encounter biotic or abiotic stress during the fig fruit development; thus the immune response would be activated and *FcJAZs* gene expression may be restricted. The findings of the present study showed that most *FcJAZs* were significantly downregulated from stage 3 to 5 in the female flower and from stage 4 to 5 in the fruit peel. These findings were consistent with the results on strawberry that most *FaJAZs* were downregulated during strawberry development and ripening [54]. WGCNA analysis of *FcJAZs* showed that FcJAZs were negatively correlated with expansin and polygalacturonase, and were positively correlated with superoxide dismutase, ascorbic acid and ficin synthesis during fig fruit development (Table S3). Similar results were reported in the rubber tree, although the direct correlation between JAZ and ficin has not been verified yet [55]. However, a significant correlation has been reported for the expression of components of COI1–JA-Ile–JAZ complex, such as high expression of HbCOI1 and abundance of HbMYC1 and HbMYC2 in latex cells [56].

The *cis*-element analysis on *FcJAZs*’ promotor regions showed that the predicted elements were correlated with the plant hormones (such as JA, ABA, ethylene and GA) and stress response (including low temperature and drought). Ethylene not only regulates plant growth and response to adversity, but researchers also focus on its regulatory mechanism on the ripening and senescence of climacteric fruits [57–59]. Fig fruit has been proved to belong to respiratory climacteric type [60]. During the ripening process, it has obvious respiratory peak and ethylene release peak, showing the late characteristics of respiratory climacteric fruit ripening [61–63]. Similar to tomato and banana, their ripening is regulated by ethylene [64]. For GA, it is found that the inhibition of GA synthesis and transcription level of response pathway is the reason for fruit shedding of San Pedro fig [41]. For non-parthenocarpy Smirna fig, exogenous treatment of GA hormone can induce parthenocarpy [65]. In addition, GA_3_ plays an important role in regulating fruit size [66, 67], and GA3 seems to inhibit the ripening of both climacteric and non-climacteric fruits, such as peach and sweet cherries [66, 68].

It was reported that the expression levels of *AtJAZ1* in Group I, *AtJAZ7* and *AtJAZ10* in Group IV, and *AtMYC2* were induced under MeJA treatment [69, 70]; and in our results, the expression level *FcJAZ1* and *FcJAZ6* from group I, *FcJAZ7-10* from group IV were up-regulated 2.43–6.96 fold after MeJA treatment, and the expression of *FcMYC2* was up-regulated 3.24 fold. *FcJAZs* of the same subfamily showed significant different regulation in response to MeJA treatment between the two analyzed tissues. This finding indicates that *FcJAZs* may play an important role in JA signaling transduction in female flowers. Most *FcJAZs* in female flower tissues responded faster to MeJA treatment compared with genes in the peels. *FcJAZ6* in subfamily I and *FcJAZ7* in subfamily IV were the most significantly upregulated. Upregulation of *FcJAZ2* in subfamily V was more significant after treatment of peels with MeJA, whereas the other *FcJAZs* were only slightly upregulated. Similar results have been reported in *Brassica oleracea* and *Camellia sinensis*, that several JAZ genes were induced after MeJA treatment [14, 71]. Findings on Ethephon or GA_3_ treatment showed that some *FcJAZs* may participate in synergistic response to these two hormone treatments, whereas some *FcJAZs* implicated in antagonism response to these two hormone treatments. In female flower tissue, *FcJAZ4* responded at the early stage to the GA_3_ treatment but responded at the later stage of Ethephon treatment. *FcJAZ6* was significantly downregulated by ethephon treatment, whereas it showed upregulation in early stages after GA_3_ treatment of female flowers.

### Potential function of FcJAZs in fig

JAZ proteins can directly interact with several transcription factors and inhibit their expression. Therefore, they effectively regulate various physiological processes by modulating expression of related transcription factors [72, 73]. In JA signal transduction pathway, transcription activator MYC2 is inhibited by binding to transcription inhibitor JAZ, which prevents the transcription of downstream genes [74]. While when JAZ protein is ubiquitinated and degraded, MYC2 is released and the transcription of downstream genes were activated. It is known that MYC2 is involved in regulating the development of leaves, roots, flowers, fruits, seeds and other organs of plants. For example in fruit development, the expression of transcription factor MYC2 was up-regulated in apple fruit treated with MeJA, and MYC2 directly combined with promoters of ACS1 and ACO1 genes and enhanced their expression, thus increasing ethylene synthesis and promoting fruit ripening [57]. MYC2 transcription factor protein plays a central role in the response to jasmonic hormones, and directly regulates downstream response genes. It has been proved that MYC2 can activate many physiological processes during fruit development, and the combination of JAZ and MYC2 would inhibit these downstream activation reactions. After JAZ degradation, MYC2 is released and the JA response is activated. In our study, we found one bHLH transcription factor FcMYC2, and Y2H analysis showed that the FcMYC2 transcription factor directly interacted with all FcJAZs members, and 8 combinations of FcJAZs-nYFP and cYFP-FcMYC2 were further proved they may have directly interaction.

Previous studies report that R2R3 MYB transcription factors can form a MBW complex with bHLH transcription factors and WD-repeat proteins [75]. Moreover, JAZ proteins can bind to bHLH or R2R3 MYB transcription factors of the MBW complex, thus inhibiting formation of the transcription complex. These implies that JAZs play an inhibiting role to the related biological processes, such as anthocyanin synthesis and epidermal hair production in plants [76]. Furthermore, the IIIf family members GL3, EGL3 and TRANSPARENT TESTA8 (TT8) of Arabidopsis interact with 8 AtJAZ proteins implicated in regulation of biosynthesis of flavonoids (anthocyanins and proanthocyanins) [76, 77]. In addition, MYB transcription factors play important roles in regulation of anthocyanins synthesis. Further, most MYB transcription factors that respond to JAs belong to the R2R3-MYB family. For example, AtMYB75 promotes accumulation of anthocyanins [76]. In the present study, WGCNA analysis of the *FcJAZs* during the fig fruit development showed that the modules containing differentially expressed genes closely related to FcJAZs were enriched in flavonoid biosynthesis, phenylpropanoid biosynthesis, flavone and flavonol biosynthesis. This implies that FcJAZs may play an important role in regulation of fig fruit coloring. There are 4 bHLH transcription factors in Arabidopsis directly interact with the JAZ repressor to inhibit JA response. Notably, bHLH3/bHLH13/bHLH14/bHLH17 tetraploid mutants showed enhanced activities in plant defenses [78]. Similar results have been reported in rice, that the OsbHLH148 interacts with OsJAZ protein leading to increased drought tolerance [79]. The findings of the current study showed one bHLH transcription factor FcMYC2. Y2H analysis showed that the FcMYC2 transcription factor directly interacted with all FcJAZs members. Notably, both *FcJAZ5* and *FcJAZ9* promotor regions contain a MYB binding site which is a regulation site (aC(G/C)GTTA) involved in regulation of flavonoid biosynthetic genes [37].

Findings on protein interaction prediction indicated that FcJAZs may play some other important roles in fig. For example, FcJAZ8 can interact with one R2R3-MYB and three WD40 proteins, which is consistent with findings that MYB24 regulates JA-mediated anther development and filament elongation by interaction with JAZs in Arabidopsis [80]. FcJAZ9 and FcJAZ10 were predicted to interact with WD40 protein of SAP, which controls organ size by targeting PPD protein degradation in Arabidopsis. WRKY40, WRKY57 and TPL interact with FcJAZs and negatively control JA-induced leaf senescence and root meristem development [72, 81]. FcJAZ1 interacted with S-phase kinase associated protein 1 (SKP1) to connect with cullin 1 (CUL1), and is implicated in mediating the response to auxin and jasmonic acid [82]. These potential functions of FcJAZs implied that they play important roles in regulation of the slow growth of fig fruits, synthesis of flavonoids, development of anthers and senescence of leaves.

## Conclusion

There are 10 FcJAZs family members were identified in fig (*Ficus carica* L.). All the members had TIFY and Jas conserved domains. Y2H analysis showed that all FcJAZs may interact with the transcription factor FcMYC2. Gene structure and *cis*-elements analysis showed that most of the promoters of *FcJAZ*s comprised various plant hormone and stress responsive related elements. Expression profiles of *FcJAZs* during fig fruit development and after treatment of fig fruit with MeJA, Ethephon and GA_3_ were analyzed. The findings showed that *FcJAZs* play an important role in fig fruit development. WGCNA analysis further showed that the expression pattern of *FcJAZs* were highly correlated with hormone signal transduction and plant-pathogen interaction. In summary, the findings of the current study provide bioinformatics basis for further functional analysis of FcJAZs to explore their roles in regulation of fig fruit development and stress resistance.

## Materials and methods

### Plant material

Six years old common fig cv. Zibao (purple peel), a bud mutation of the most important fig cultivar Qingpi (green peel) in China, was used as the experimental material in this study, which planted in Weihai city, Shandong Province, China (37°25′ N, 122°17′ E). Professor Huiqin Ma, the co-author of this study, undertook the formal identification of this cultivar [29] and obtained the variety certificate issued by the State Forestry Administration of China (Plant Breeder’s Right Number: 20150145). We obtained the permission to collect fig fruits. This study did not involve the collection of plant specimens for storage.

### Identification of JAZ family genes in fig (*Ficus carica* L.)

The first phase of identification of candidate JAZ family members, Hidden Markov Model (HMM) profiles of TIFY domain (Pfam: PF06200) and jas domain (Pfam: PF09425) were retrieved from Pfam (http://pfam.xfam.org/). These domains were used for protein screening using HMMER 3.2.1 (e-value < 0.01) based on the whole-genome protein data of “Horaishi” and “Dottato” (https://www.ncbi.nlm.nih.gov/genome/?term=Ficus+carica) [35, 36]. The second phase of identification of candidate JAZ family members involved retrieval of JAZ genes of mulberry and Arabidopsis from MorusDB (http://morus.swu.edu.cn/index.html/) and Arabidopsis Hormone Database (AHD) v2.0 (http://ahd.cbi.pku.edu.cn/) [83]. Identification of non-redundant predicted protein sequences in the fig genomes was conducted using BLASTP program with Arabidopsis and mulberry JAZs protein sequences as query sequences [84]. Genes annotated as JAZ proteins were searched based on RNA-seq database annotations, candidate genes were manually merged and repetitive sequences were deleted to obtain a complete family member library. Candidate JAZ proteins were further analyzed for identification of conserved domain using Pfam and NCBI-CDD (https://www.ncbi.nlm.nih.gov/Structure/cdd/wrpsb.cgi) databases.

### Analysis of sequence characteristics and phylogenetic tree construction

JAZs protein sequences were analyzed by Clustal X by [85] and submitted to MEME (http://meme-suite.org/) for motif prediction. MEME motif sequence length was set to 20–100 aa, maximum number of discoveries was set to 4, and other parameters were set to default values. Alignments were visualized and edited with Jalview tool [86]. To explore intron–exon organization of the *JAZs* genes, coding sequences with corresponding genomic sequences were aligned. The results retrieved using the Gene Structure Display Server (http://gsds.cbi.pku.edu.cn/index.php). The upstream 2000 bp regions of *FcJAZs* were considered to have the full length promoters. Regulatory *cis*-elements were predicted using PlantCARE tool (https://bioinformatics.psb.ugent.be/webtools/plantcare/html/) and PlantPAN tool (https://plantpan.itps.ncku.edu.tw/). The theoretical isoelectric point (pI) and molecular weight (Mw) of each JAZ protein were determined using the ‘Compute pI/Mw tool’ in ExPASy (https://web.expasy.org/compute_pi/). Subcellular locations were predicted using WoLFPSORT (https://psort.hgc.jp/cgi-bin/runpsort.pl). Chromosomal position of the *FcJAZ* family genes were obtained based on the genomic sequence information and maps were constructed using Map Gene 2 Chromosome V2 tool (http://mg2c.iask.in/mg2c_v2.0/). Protein sequences were aligned using ClustalW program and phylogenetic trees were constructed using the Neighbor-Joining (NJ) and method in MEGA6 (http://www.megasoftware.net/) and the bootstrap value was set to 1000. The phylogenetic tree of FcJAZ genes and two species (AtJAZ and MnJAZ) was also constructed using the Maximum Likelihood (ML) method in MEGA 6, and the Jones-Taylor-Thornton (JTT) was selected as the model.

The non-synonymous replacement rate (Ka) and synonymous replacement rate (Ks) of the replicated gene pairs were calculated using KaKs_Calculator 2.0, and environmental selection pressure was analyzed by Ka/Ks ratio.

### Analysis of interactive signaling network

Protein interaction network (interactome) for JAZ-JAZ proteins was constructed using STRING tool (http://string-db.org/). After analysis of the relationship between proteins using the protein interaction network, networkX was using in Python environment to visualize the network of genes of interest. Protein–protein interaction networks were built with an interaction score of the highest confidence (0.900); the minimum required interaction score was 0.150 (was considered as low confidence).

### Yeast two-hybrid assay and bimolecular fluorescence complementation assay

The full-length sequences of all *FcJAZs* were cloned into the pGAD-T7-AD vector, whereas the full-length sequences of *FcMYC2* and *FcbHLH33* were cloned into the pGBK-BD vector following a protocol by Matchmaker™ Gold Yeast Two-Hybrid system (Clontech, Mountain View, CA, USA). Primers used for Y2H are listed in Table S1. Yeast AD/BD co-transformations were performed using lithium-acetate method [87]. Interactions between proteins were determined by growth on the DDO medium (SD/–Trp/–Leu/) at 30 °C. After growing the plaque, they were transferred to the QDO medium (SD/–Trp/–Leu/–His/–Ade) containing 5-bromo-4-chloro-3-indoxyl α-D-galactoside (X-α-Gal) and incubated for three days at 30 °C.

To co-transform the paired combination of FcJAZs and FcMYC2, the vectors used are pSPYNE-35S and pSPYCE-35S, and the primers used are listed in Table S1. After infection, the tobacco was cultured in the dark for 24 h, and then grown for 1 day under the conditions of 16 h of light, 8 h of darkness and 25 ℃. Cut the infected leaves and place them on a microscope slide. Use a confocal microscope to observe the fluorescence of YFP (trigger wavelength is 514 nm, radiation wavelength is 527 nm). BiFC experiments were repeated at least two times in independent experiments.

### Plant growth conditions and treatments

Common fig cv. Zibao fruits were harvested from Shandong province, P. R. China. Six stages of autumn fruit were sampled for gene expression analysis based on the characteristics of fruit development. The stages were marked as stage 1–6, whereby stage 2, 3, and 4 were the early, middle and late stages of phase II (slow growth period), stage 1 represented phase I (fast growth period), stage 5 and 6 represented phase III (fast growth period). Sixty fruits were randomly selected at each stage and 20 fruits were used as a biological replicate. The peel and female flower tissue were separated at the sampling time. Fresh samples were quick-frozen with liquid nitrogen and stored at -80 °C for subsequent experiments.

The peel and female flower tissue of the stage 3 fruit were obtained for MeJA treatment. The separated tissues were immersed in 200 μM MeJA (sample: MeJA solution = 1:3, w/w), shaking was performed at 25 °C at 30 rpm under dark conditions. The control was treated with the dissolving solution (1% ethanol solution) without MeJA and shaken under the same conditions. Untreated samples were collected and labeled as 0 h. After 1 h, 3 h, 9 h, 24 h of shaking, the solution on the surface of the samples was absorbed using a filter paper, then frozen with liquid nitrogen and stored at -80 °C for subsequent experiments.

### Expression pattern analysis during fig fruit development

Expression patterns of *FcJAZ* genes in the female flower and peel tissue during fig fruit development were explored. Autumn fig fruit female flower and peel were obtained from Zibao fruit at the six stages as mentioned above and were used for RNA-seq analysis. Data were retrieved from the NCBI database (NCBI No. PRJNA723733). Stage 1 samples were collected from phase I of the rapid growth period of fig whereas stage 2, 3, and 4 samples were obtained from the early, middle and late stages of the slow growth of period II of fig, respectively; stage 5 and 6 were located in the early and late stages of the second rapid growth (period III) of fig. To explore expression patterns of *FcJAZ* genes after different hormone treatments, RNA-seq data of the fig transcriptome after treatment with Ethephon and GA_3_ were analyzed also analyzed. Transcriptome data were obtained from findings of a related research by our team (Accession No. SRP113799 and No. PRJNA606407) [41, 42]. For RNA-seq analysis, fragments per kb per million (FPKM) method was used to normalize and determine gene expression levels of JAZ and JAs-related genes in different treatments after hormone application (Wang et al., 2010).

### Co-expression network analysis during fig fruit development

Weighted gene co-expression network analysis (WGCNA) [88] was used to analyze gene co-expression network during the fig fruit development. The threshold power was set to nine. The network included phenotypic data, based on filtered data and sample size. Correlations were determined using Pearson’s correlation analysis. The common expression module was identified, and the minimum connection value, module merging standard, and minimum module size were set to 0.5, 0.25, and 30, respectively. After identifying the co-expression modules, the modules were associated with each sample type. DESeq2 was used to identify differentially expressed genes (DEGs) (∣log_2_(Fold Change)∣ > 1, *p* value < 0.05) [89]. Gene ontology analysis was performed using Kyoto Encyclopedia of Genes and Genomes (KEGG) database [90].

### Quantitative RT-PCR (qRT-PCR)

Fig fruit RNA was extracted with the CTAB method [91]. RNA concentration and purity were determined by NanoDrop 2,000 (Thermo Scientific, USA). 1% agarose gel electrophoresis was performed to determine RNA integrity, then the RNA concentration was adjusted to a consistent level (1,000 ng). First strand cDNA was synthesized using cDNA Synthesis Kit (Takara, Kyoto, Japan). Ultra SYBR Mix kit (Takara, Kyoto, Japan.) was used to perform qRT-PCR using the ABI Q6 real-time PCR system (Applied Biosystem Inc., MA, USA). The reaction volume of PCR was 30 ml which comprised 3 L diluted cDNA, 3 L of each primer (2 mM), 15 L of SYBR Green Realtime PCR Master Mix Plus and 3 l Plus solution (Toyobo). PCR amplification was initiated by 1 min at 95 °C, followed by 40 cycles of 95 °C for 15 s, 58 °C for 15 s and 72 °C for 45 s. All experiments were performed in triplicate using diluted cDNAs. Gene-specific primers listed in Table S1 were used for qRT-PCR. Relative quantitative analysis of data was performed by the 2^−ΔΔCT^ method with reference genes *Fcactin* and *18S-RNA*. Three technical replicates were carried out for each sample to ensure reproducibility and reliability. Statistical analysis of variance (ANOVA) followed by Duncan’s new multiple range test and Student’s *t* test were performed with SPSS Version 16.0 (Chicago, IL, USA). The significance level was set to *P* < 0.05.

## Supplementary Information

**Additional file 1:** **Figure S1. **Multiple sequence alignment of the full-length JAZ proteins from fig, mulberry and Arabidopsis. The alignments of the JAZ protein sequences were performed by CLUSTALW. Red residues indicated the conservation of amino acid identity were at least 50% of the aligned proteins, whereas residues conserved in all protein sequences were highlighted in red-shaded. The conserved sequence of the motif of TIFY, Jas and EAR were indicated at the bottom of the relevant place

**Additional file 2: Figure S2. **Differentially Co-expressed genes of *FcJAZs *between the fig female flower and peel tissue. The RNA-seq data for the fig fruit development were used for the WGCNA analysis, see the details in Fig 4. (**a**) Venn diagram showed the shared and unique Differentially Expressed Genes (DEGs) in the fig female flower and peel tissue, which Co-expressed with the *FcJAZs*. KEGG analysis suggested that the DEGs with the consistent regulation trend between tissues were represented in (**b**) and the different regulation trend between tissues were represented in (**c**) 

**Additional file 3:** **Figure S3. **Multiple sequence alignment of the full-length MYC2 proteins**(a)**The conserved domains in FcMYC2 which were detected by NCBI Conserved Domains Search. **(b) **The details of these proteins’ sequence information in the NCBI database were that, FcMYC2 (c5167_g1), MnMYC2 (XP010104300.1), AtMYC2 (At1g32640.1), CsMYC2 (XP030501877.1), VvMYC2 (XP002280253.1), OsMYC2 (XP015614012.1), SlMYC2 (NP001311412.1), AtMYC3 (At5g46760.1) and AtMYC4 (At4g17880.1). The alignments of the JAZ protein sequences were performed by CLUSTALW

**Additional file 4:** **Figure S4.** The interaction network of FcJAZs according to the orthologues in Arabidopsis. This network was predicted by online software STRING. FcJAZ protein was shown by gene ID. The cluster was generated using Kmeans clustering algorithm from STRING database. Different colors indicate different clusters. Red boxes indicate proteins that were predicted to interact with FcJAZ1, FcJAZ8, FcJAZ9 and FcJAZ10 proteins

**Additional file 5:** **Figure S5. ***BiFC*visualization of FcJAZs and FcMYC2 interactions in tobacco leaf cells. FcJAZs and FcMYC2 were fused with the N- and C-termini of YFP and all ten possible interactions were tested. The interactions between FcMYC2 fused with N-terminal YFP and empty vector with C-terminal YFP were tested as negative controls. The *mCherry*carrying a nuclear localization signal was used as the nuclear marker. Bars=50 μm

**Additional file 6:** **Table S1. **Primers sequence used in amplification, qPCR, yeast one-hybrid and BiFC

**Additional file 7:** **Table S2. **Promoter analysis of the Fig JAZ gene family

**Additional file 8:** **Table S3. **WGCNA of JAZs related to different metabolites

**Additional file 9:** **Table S4. **Ka/Ks analysis for the JAZ duplicated genes

**Additional file 10:** **Table S5. **KEGG enrichment analysis of co-expression patterns genes in WGCNA modules

## Data Availability

The RNA-Seq data have been deposited in NCBI (SRA accession: PRJNA723733, SRP113799 and No. PRJNA606407).

## Abbreviations

ABA: Abscisic acid
AbA: Aureobasidin A
Ade: Adenine
AHD: Arabidopsis Hormone Database
Bp: Base pair
cDNA: Complementary DNA
COI1: Coronatine insensitive 1
CTAB: Cetyl triethylammol/lonium bromide
DDO: Double dropout SD agar plate
DEGs: Differentially expressed genes
EAR: ERF-associated amphiphilic repression
EIL1: EIN3-LIKE1
EIN3: ethylene insensitive3
FPKM: Fragments per kilobase of transcript per million fragments mapped
GA: Gibberellin
GO: Gene ontology
His: Histidine
HMM: Hidden Markov Model
JA: Jasmonic acid
JA-Ile: Jasmonoyl-isoleucine
Jas: Jasmonates
JAZ: Jasmonate-ZIM
KEGG: Kyoto Encyclopedia of Genes and Genomes
Lue: Leucine
MeJA: Methyl jasmonate
MEME: Multiple Em for Motif Elicitation
Mw: Molecular weight
NINJA: Novel interactor of JAZ
NJ: Neighbor-Joining
PCR: Polymerase Chain Reaction
pI: Isoelectric point
QDO: Quadruple dropout SD agar plate
qRT-PCR: Fluorescence quantitative real-time
PCR; rmp: Revolutions per minute
RNA-Seq: RNA transcriptome sequencing
SCFCOI1: Skip/Cullin/F-box-type
TPL: TOPLESS
Trp: Tryptophan
WGCNA: Weighted-gene co-expression network analysis
X-α-Gal: 5-bromo-4-chloro-3-indolyl-α-D-galactoside
Y2H: Yeast two hybrid
