# Supplementary figures and images for: Genome-wide analysis of JAZ family genes expression patterns during fig (Ficus carica L.) fruit development and in response to hormone treatment
Source: BMC Genomics. 2022 Mar 2;23:170. doi: 10.1186/s12864-022-08420-z (PMC8889711; doi:10.1186/s12864-022-08420-z)

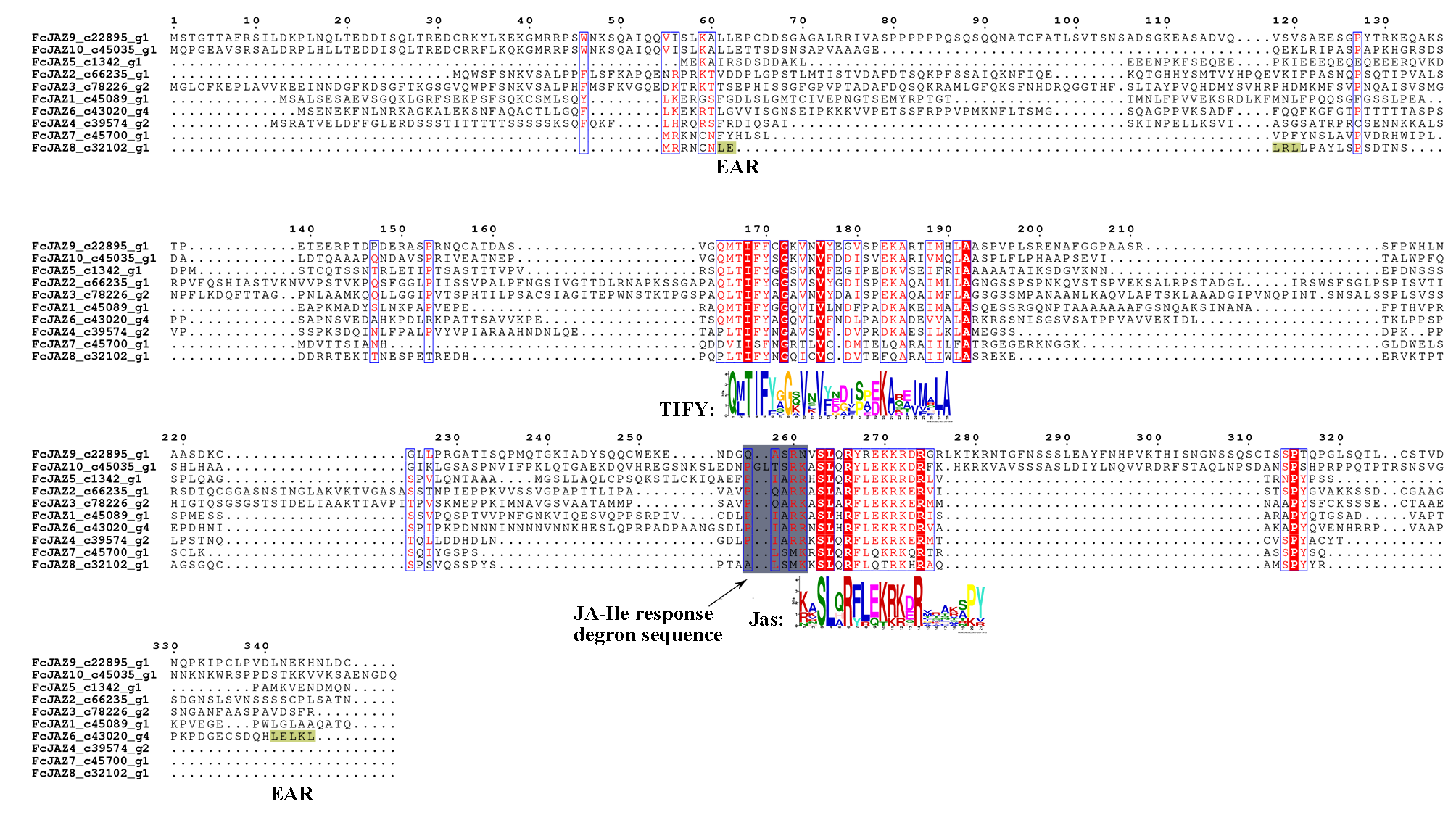

Supplement: Supplementary file 1 — Additional file 1: Figure S1. Multiple sequence alignment of the full-length JAZ proteins from fig, mulberry and Arabidopsis. The alignments of the JAZ protein sequences were performed by CLUSTALW. Red residues indicated the conservation of amino acid identity were at least 50% of the aligned proteins, whereas residues conserved in all protein sequences were highlighted in red-shaded. The conserved sequence of the motif of TIFY, Jas and EAR were indicated at the bottom of the relevant place [file 12864_2022_8420_MOESM1_ESM.tif]

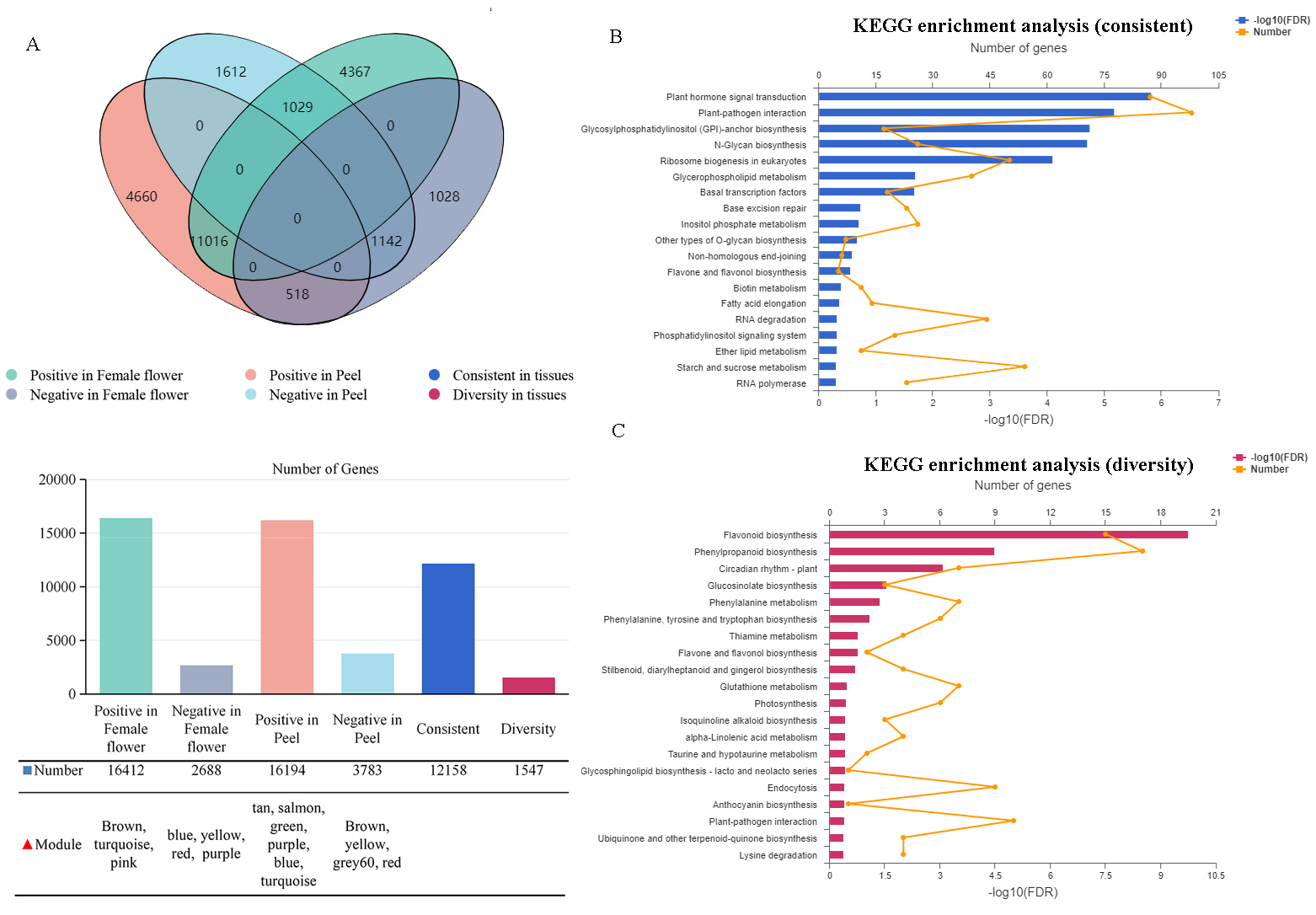

Supplement: Supplementary file 2 — Additional file 2: Figure S2. Differentially Co-expressed genes of FcJAZs between the fig female flower and peel tissue. The RNA-seq data for the fig fruit development were used for the WGCNA analysis, see the details in Fig 4. (a) Venn diagram showed the shared and unique Differentially Expressed Genes (DEGs) in the fig female flower and peel tissue, which Co-expressed with the FcJAZs. KEGG analysis suggested that the DEGs with the consistent regulation trend between tissues were represented in (b) and the different regulation trend between tissues were represented in (c) [file 12864_2022_8420_MOESM2_ESM.tif]

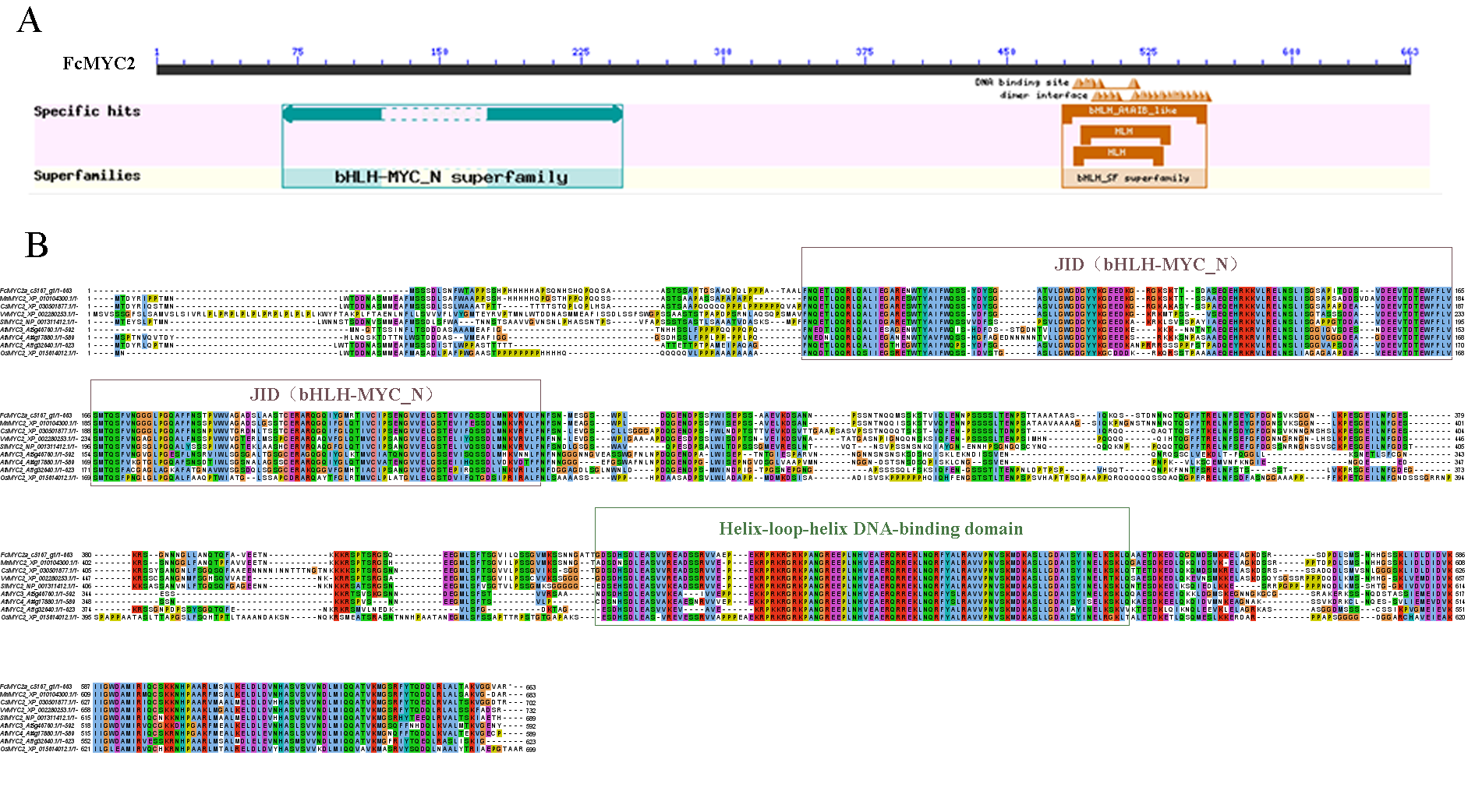

Supplement: Supplementary file 3 — Additional file 3: Figure S3. Multiple sequence alignment of the full-length MYC2 proteins(a)The conserved domains in FcMYC2 which were detected by NCBI Conserved Domains Search. (b) The details of these proteins’ sequence information in the NCBI database were that, FcMYC2 (c5167_g1), MnMYC2 (XP010104300.1), AtMYC2 (At1g32640.1), CsMYC2 (XP030501877.1), VvMYC2 (XP002280253.1), OsMYC2 (XP015614012.1), SlMYC2 (NP001311412.1), AtMYC3 (At5g46760.1) and AtMYC4 (At4g17880.1). The alignments of the JAZ protein sequences were performed by CLUSTALW [file 12864_2022_8420_MOESM3_ESM.tif]

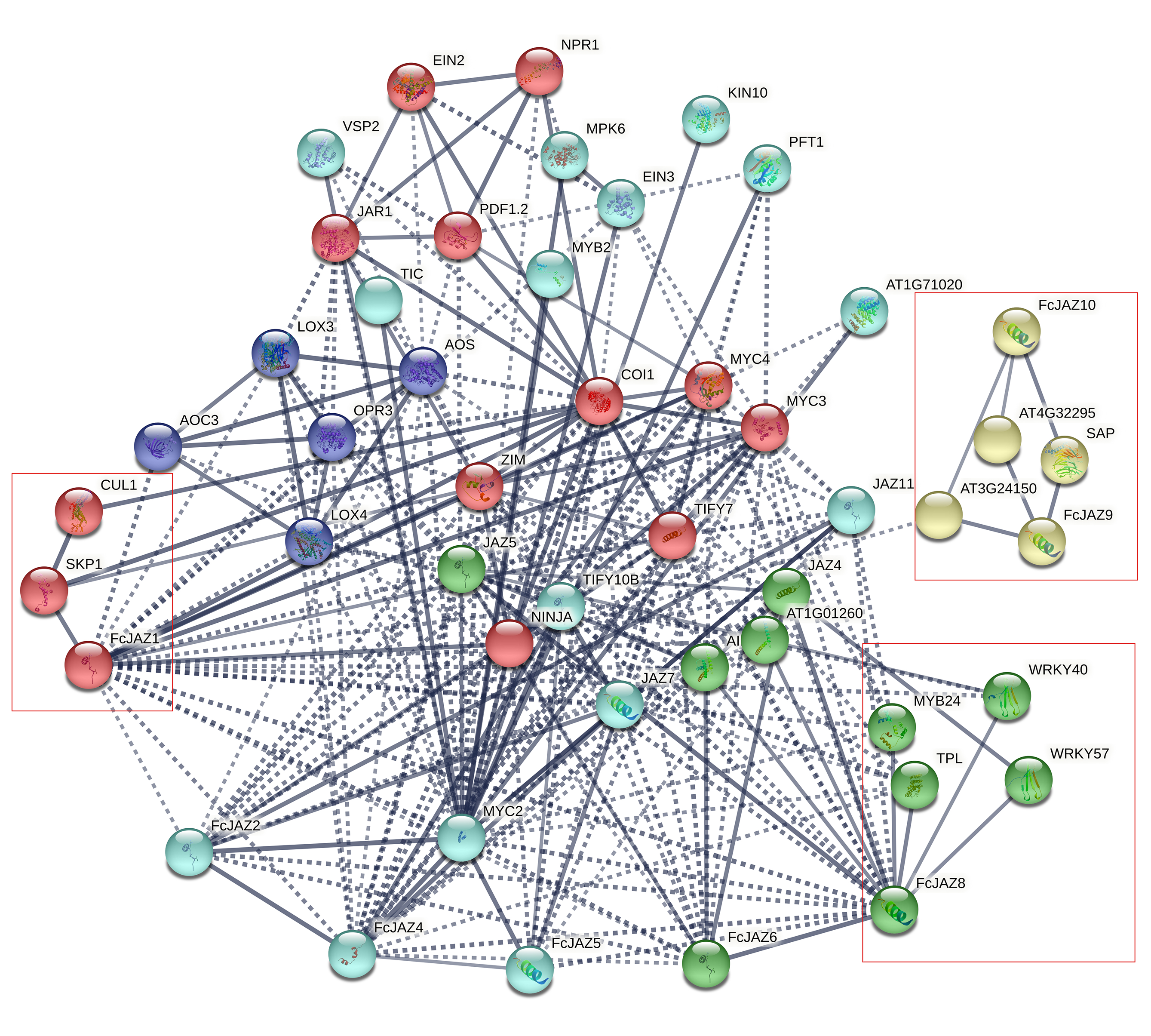

Supplement: Supplementary file 4 — Additional file 4: Figure S4. The interaction network of FcJAZs according to the orthologues in Arabidopsis. This network was predicted by online software STRING. FcJAZ protein was shown by gene ID. The cluster was generated using Kmeans clustering algorithm from STRING database. Different colors indicate different clusters. Red boxes indicate proteins that were predicted to interact with FcJAZ1, FcJAZ8, FcJAZ9 and FcJAZ10 proteins [file 12864_2022_8420_MOESM4_ESM.tif]

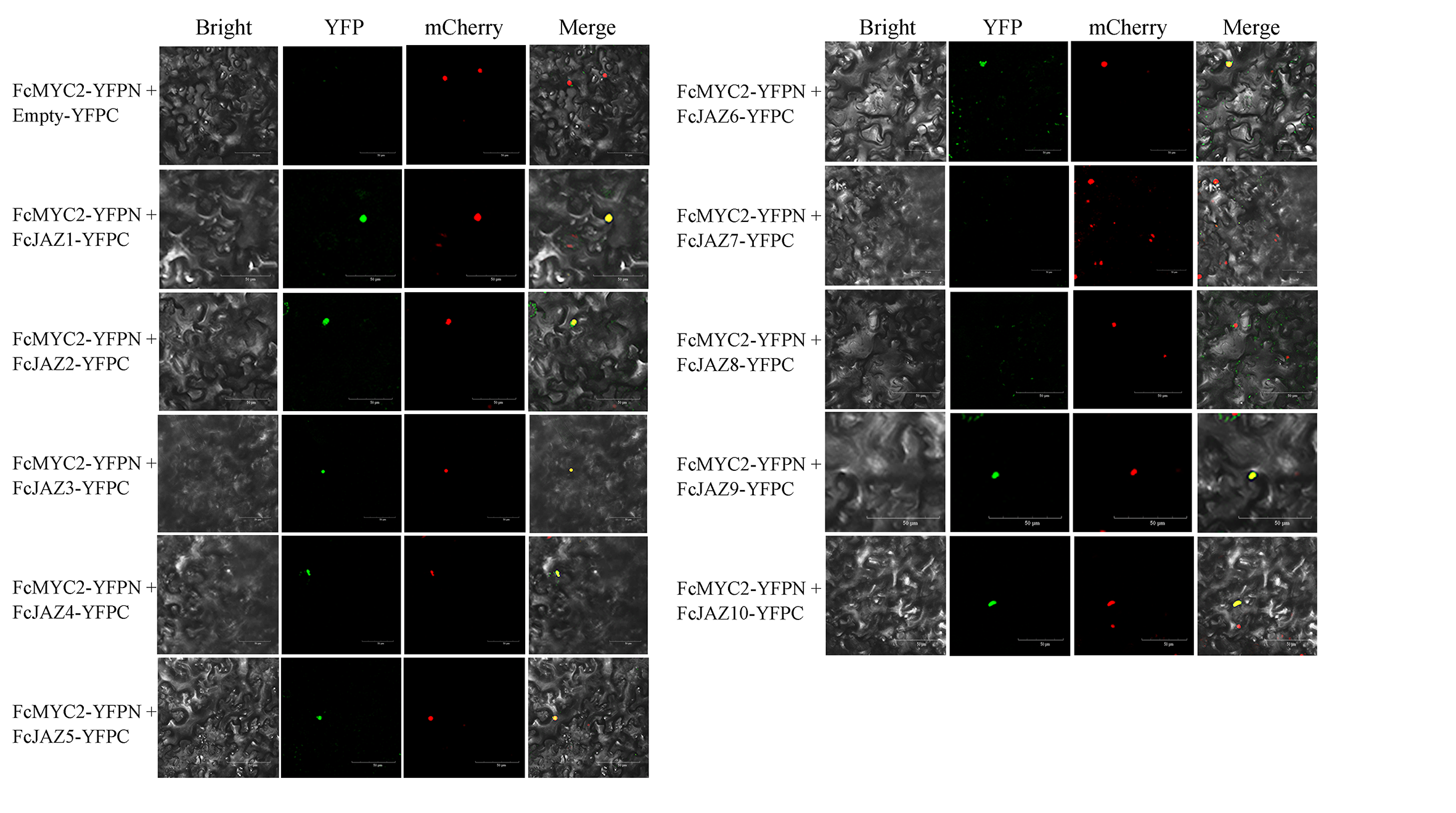

Supplement: Supplementary file 5 — Additional file 5: Figure S5. BiFCvisualization of FcJAZs and FcMYC2 interactions in tobacco leaf cells. FcJAZs and FcMYC2 were fused with the N- and C-termini of YFP and all ten possible interactions were tested. The interactions between FcMYC2 fused with N-terminal YFP and empty vector with C-terminal YFP were tested as negative controls. The mCherrycarrying a nuclear localization signal was used as the nuclear marker. Bars=50 μm [file 12864_2022_8420_MOESM5_ESM.tif]
